# Dietary Habits and Physical Activity of 18-Year-Old Adolescents in Relation to Overweight and Obesity

**Published:** 2019-05

**Authors:** Magdalena ZALEWSKA, Elżbieta MACIORKOWSKA

**Affiliations:** 1. Department of Public Health, Medical University of Bialystok, Szpitalna 37, 15-295 Bialystok, Poland; 2. Department of Developmental Medicine and Pediatric Nursing, Medical University of Bialystok, Szpitalna 37, 15-295 Bialystok, Poland

**Keywords:** Adolescents, Nutrition status, Diet, Physical activity, Sedentary behaviors

## Abstract

**Background:**

The purpose of this study was to evaluate the correlations between the nutritional status, nutritional habits, and physical activity in the representative population of 18-year-old students.

**Methods:**

A total of 1,631 participants aged 18 years, originating from secondary schools/senior high schools in the city of Bialystok, Szpitalna, Bialystok, Poland were enrolled. Participants completed anonymous questionnaires, and their body mass index was assessed. Data were analyzed using standard statistical procedures with Statistical PL 12.0.

**Results:**

The analysis showed that consumption of only one to two or three meals was associated with an increased risk of overweight and obesity—in case of one or two meals among girls, OR=1.78 (*P*<0.05), in case of one or two meals among boys, OR=2.325 (*P*<0.01), and in case of three meals, OR=1.771 (*P*<0.05). First breakfast was consumed by 82.1% of boys with normal BMI and 73.1% overweight and obesity (OR=0.593; *P*<0.05). 24.5% of the eutrophic compared to only 20.3% of adolescents with elevated BMI reported attending all PE classes. Physical activity undertaken after school was associated with a 35.9% decrease risk of overweight and obesity among girls and 57.7% among boys.

**Conclusion:**

A specific pattern of nutritional habits, that is, decreased meal frequency in adolescents, skipping meals, appear the most frequent nutritional mistakes associated with overweight and obesity. Insufficient physical activity and exposure to sedentary behaviors among adolescents are disturbing. The obesity problem requires monitoring, prevention and the change of nutritional habits and physical activity early in childhood.

## Introduction

Nutritional requirements during the growth of children and adolescents are regarded as critical factors affecting their physical development and also influencing their health status in the future life. It has been shown that both nutritional habits and nutrients intake may, at least partly, prevent some chronic civilization diseases such as obesity, atherosclerosis, hypertension, or osteoporosis (1–3).

Obesity, including its increasing worldwide prevalence of childhood obesity, is considered a major public health problem not only in the developed countries, but also in the developing regions of the world. Although the underlying mechanisms of obesity epidemics among children and adolescents are complex, there are still two important pathogenic factors responsible for excessive weight gain during growth: nutritional habits and inadequate physical activity. An increased caloric intake leading to positive energy balance is usually the result of inappropriate dietary regimen (e.g., over-eating, irregular food consumption or meal frequency, diets rich in simple carbohydrates, more fat, especially saturated fats, and fiber deficits) as well as low levels of physical activity. Therefore, the principle of obesity management in adolescence is targeted to achieve negative energy balance, both quantitative and qualitative dietary modifications, and a considerable increase in physical/sports activities (4–6).

The purpose of this study was to evaluate correlations between the nutritional status, nutritional habits, attitudes toward recommended daily food consumption, and physical activity in the representative population of 18-year-old students. The study was aimed at factors potentially associated with overweight and obesity in this population.

## Materials and Methods

A total of 1,660 subjects, aged 18 years, originating from secondary schools/senior high schools randomly selected in the city of Bialystok, were included in the study. Due to strict chronological age exclusion criteria and drop-off rate (invalid questionnaire response), 1,631 participants (girls N=1,051, 64.5%; boys N=580, 35.5%) were qualified for further analyzes. Both participants and school authorities gave their informed written consent for the study and approved protocol. An original anonymous supervised questionnaire was distributed among participants between September and November 2010.

The validation of the questionnaire was initially carried out in a group of 60 age-matched students attending schools in the urban area of Bialystok. Body weight and height were measured using an electronic scale (Seca) and Holtain anthropometer, the results were compared with the updated national reference, and body mass index (BMI) was calculated for each individual in a standard way with the World Health Organization (WHO) criteria. The standard percentile charts for 18-year-old girls and boys were used to interpret the categories of normal BMI and obesity, and the data distribution including the obesity rate was evaluated. BMI < 5 percentile indicated thinness, and BMI ≥ 85 percentile as overweight and obesity. BMI values in between 5 and 85 percentiles referred to normal weight of youth. For further analysis of nutritional status and dietary behaviors, BMI values were recorded for normal weight, overweight, and obese students.

The questionnaire included questions about food behaviors, main meal frequencies, physical activity, sedentary behaviors, and family socioeconomic status.

These data were analyzed using standard statistical procedures with Statistica PL 12.0 (StatSoft Poland). The logistic regression model was applied to estimate the effect of physical activity and dietary habits on nutritional status. The results of analysis were presented as odds ratios (OR) with 95% confidence interval (95% CI) and *P*-values of the Wald’s test.

The study was approved by the Ethical Committee of the Medical University of Bialystok.

## Results

Mean height of girls was 167.5 ± 6.0 cm and weight was 58.0 ± 8.4 kg. Mean height of boys was 181.3 ± 6.5 cm and weight was 73.4 ± 10.3 kg. Mean BMI values of girls and boys ranged from 16.0 to 42.5 (mean 20.6 ±2.5) and from 16.9 to 33.2 (mean 22.3 ±2.6), (Table 1). Of all the studied participants, 78.4% of girls and 78.0% of boys (N=1,275) represented eutrophic status (BMI values ranged from 5^th^ to 85^th^ percentiles). Inadequate nutritional status reflecting underweight (i.e., BMI below 5 percentiles) was found in 10.5% of girls and 4.1% of boys (N=135). Overweight and obesity were found in 11.1% of girls and 17.9% of boys (N=221).

**Table 1:** Anthropometric characteristics of male and female participating in the study

***Anthropometric parameters***	***Mean ± SD***
**Girls (n=1051)**	
Body weight [kg]	58.0±8.4
Body height [cm]	167.5±6.0
BMI	20.6±2.5
**Boys (*n*=580)**	
Body mass [kg]	73.4±10.3
Body weight [cm]	181.3±6.5
BMI	22.3±2.6

Based on the analysis of the number of meals usually consumed by these adolescents, we demonstrated that as many as 29.9% of overweight and obese girls and 23.0% boys had one or two meals compared to normal-weight adolescents (21.6% and 14.2%). The recommended number of meals, that is, four or five meals, was consumed by 33.3% of obese girls and by 42.9% of normal-weight girls. A total of 54.8% of normal-weight boys and 38.5% of the obese consumed four or five meals. The analysis showed that consumption of only one to two or three meals was associated with an increased risk of overweight and obesity in case of one or two meals among girls, OR=1.78 (*P*<0.05); in case of one or two meals among boys, OR=2.325 (*P*<0.01), and in case of three meals, OR=1.771 (*P*<0.05) (Table 2).

**Table 2:** Meal frequency and regularity in relation to BMI in studied adolescents

***Variable***	***Girls***							***Boys***						
	***5–85 BMI percentile***		***>=85 BMI percentile***		***OR***	***P value***	***95% CI***	***5–85 BMI percentile***		***>=85 BMI percentile***		***OR***	***P value***	***95% CI***
	***N=823***	***%***	***N=117***	***%***				***N=452***	***%***	***N= 104***	***%***			
Number of meals:														
4–5 *(reference category)*	353	42.9	39	33.3	–	0.07	–	248	54.8	40	38.5	–	0.007	–
3	292	35.5	42	36.8	1.333	0.221	0.841–2.112	140	31.0	40	38.5	1.771	0.021	1.091–2.876
1–2	178	21.6	35	29.9	1.78	0.021	1.090–2.907	64	14.2	24	23.0	2.325	0.004	1.307–4.135
Daily meals:														
Breakfast														
Yes	650	79.0	88	75.2	0.808	0.354	0.514–1.269	371	82.1	76	73.1	0.593	0.038	0.361–0.973
No	173	21.0	29	24.8				81	17.9	28	26.9			
Lunch														
Yes	480	58.3	56	47.9	0.656	0.033	0.445–0.967	241	53.3	47	45.2	0.722	0.136	0.471–1.108
No	343	41.7	61	52.1				211	46.7	57	54.8			
Supper														
Yes	596	72.4	77	65.8	0.733	0.139	0.486–1.106	415	91.8	87	83.6	0.456	0.013	0.246–0.847
No	227	27.6	40	34.2				37	8.2	17	16.4			
Regular meals:														
Never	167	20.3	35	29.9	5.868	0.087	0.773–44.577	61	13.5	25	24.1	2.004	0.111	0.852–4.711
Sometimes	387	47.0	43	36.7	3.111	0.271	0.413–23.438	176	38.9	35	33.6	0.972	0.945	0.435–2.171
Frequently	241	29.3	38	32.4	4.415	0.15	0.583–33.407	171	37.8	35	33.6	1.001	0.999	0.448–2.236
Always *(reference category)*	28	3.4	1	0.9	–	0.033	–	44	9.7	9	8.7	–	0.072	–

A significant difference was found in the nutritional status among boys in relation to the consumption of first breakfast. In this study, 82.1% of boys with normal BMI and 73.1% of those with overweight and obesity consumed first breakfast every day (OR=0.593; *P*<0.05). Among girls, 58.3% with normal BMI and 47.9% who were overweight and obese reported consuming lunch with OR being 0.656 (*P*<0.05; Table 2).

Irregularity in meals was found to be the highest among girls with overweight and obesity (29.9%) compared to eutrophic girls (20.3%). We did not find similar correlation among boys (Table 2).

The patterns of physical activity based on sports and training scheduled for physical education (PE) classes at school and after-school outdoor activities are shown in Table 3.

**Table 3:** Physical activity in eutrophic, overweight, and obese adolescents

***Variable***	***Girls***							***Boys***						
	***5–85 BMI percentile***		***>=85 BMI percentile***		***OR***	***P Value***	***95% CI***	***5–85 BMI percentile***		***>=85 BMI percentile***		***OR***	***P value***	***95% CI***
	***N=823***	***%***	***N=117***	***%***				***N=452***	***%***	***N= 104***	***%***			
Participation in the classes of physical education (PE)														
Always *(reference category)*	161	19.5	19	16.2	–	0.730	–	151	33.4	26	25.0	0.286	–	–
Regularly	456	55.4	64	54.7	1.189	0.531	0.691–2.046	224	49.6	56	58.9	1.452	0.151	0.873–2.415
Irregularly	101	12.3	16	13.7	1.342	0.416	0.660–2.730	43	9.5	10	9,6	1.351	0.464	0.604–3.018
Long-term dismissal from PE classes	105	12.8	18	15.4	1.453	0.289	0.729–2.896	34	7.5	12	11.5	2.050	0.071	0.941–4.465
Systematic physical activity after school														
Yes	367	44.6	42	35.9	0,696	0.077	0.465–1.040	315	69.7	60	57.7	0.593	0.019	0.383–0.919
No	456	55.4	75	64.1				137	30.3	44	42.3			
Time spent TV watching														
<2h	500	60.8	75	64.1	1.525	0.487	0.579–1.297	313	69.2	62	59.6	0.869	0.059	0.983–2.368
≥2h	323	39.2	42	35.9				139	30.8	42	40.4			
Time spent computer using														
<2h	411	49.9	50	42.7	0.789	0.145	0.904–1.976	98	21.7	27	26.0	1.337	0.346	0.483–1.291
≥2h	412	50.1	67	57.3				354	78.3	77	74.0			

Boys reported more physical activity than girls. A total of 33.4% of the eutrophic boys compared to only 25.0% of adolescents with high BMI boys reported attending all PE classes, whereas 19.5% of eutrophic girls and 16.2% of the overweight and obese girls participated in nearly all the classes. A long-term dismissal from PE classes was reported by 10.9% of adolescents with normal BMI (12.8% girls and 7.5% boys) and by 13.6% of individuals with overweight and obesity (15.4% vs. 11.5%).

Physical activity undertaken after school was associated with a 69.6% decrease in the risk of overweight and obesity among girls and 59.3% among boys. Girls reported more hours of sedentary behavior than boys. There were no significant time differences between watching TV and using computer among girls with normal BMI and high BMI. Higher prevalence of obesity was reported by boys who were watching television for more than 2 hours a day (Table 3).

## Discussion

Our study shows a positive relationship between the number of meal consumption and higher anthropometric characteristics in adolescents. Our findings are consistent with other reports demonstrating that dividing a daily food portion into four or five meals decreases the risk of overweight and obesity among adolescents (7–10). In our study, obese students more frequently had eaten one or two meals a day compared to their peers.

According to a cross-sectional nutritional study (11), both obese girls and boys reduced the number of meals per day, skipped the breakfast, and avoided sweets and salty snacks, which are more evident in girls, as a method to counteract the obesity. Consuming the first breakfast enables to maintain appropriate body weight, whereas avoiding the first breakfast to limit energy consumed during the day may lead to consuming more meals later in a day (12–15). Moreover, the consumption of breakfast provides a higher intake of valuable nutrients in a diet, including cereals, fruit, vegetables, and dairy products (16).

Sandercock et al. (17), during the examination of 4,326 children aged 10–16 years, proved a correlation between skipping breakfast consumption and higher BMI. Other well-designed studies also confirmed these results (13, 14, 18). We found similar associations showing that obese individuals less frequent ate their first breakfast compared to eutrophic adolescents. These differences were particularly evident among obese boys and those with proper body mass. A lower proportion of obese adolescents consumed a meal during breaks between classes at school in comparison with normal BMI students. These differences were particularly evident among obese girls and those with proper body mass.

Some studies suggest that skipping breakfast is associated with unhealthy behaviors such as a lower level of physical activity (4, 19, 20). In general, adolescents, especially girls, believe that skipping breakfast could be an effective method of dieting to lose weight and reduce daily energy intakes (21). Several studies report that breakfast skipping is associated with dieting practices in girls, because of concerns about body weight and dissatisfaction with their body shape (22). Moreover, low frequencies of breakfast, lunch, and evening meal in early adolescence predicted low meal frequencies in early adulthood (23). Kann et al. (24) in a nationally representative survey of US high school students in 2013 reported that girls more often engage in unhealthy weight-control behaviors than boys, such as poorer dietary intake and less frequent meals.

In general, avoiding regular meals, consuming more calories by increasing portions, and a coincident snacking between meals may contribute to development of obesity. This is directly associated with consumption of higher amount of food and highly caloric fast-food (4). Snacking patterns increase the daily intake of calories coming from snacks and may play an important role in childhood obesity (25).

Epidemiological data indicated that physical activity decreased during puberty (26). Among adolescents examined within the program Health Behavior in School-aged Children (HBSC) in the years 2009–2010 (26), a decrease in physical activity was reported specifically in older age groups. In this critical period, the most spectacular decrease in physical activity was reported among children aged 11 years; spontaneous willingness to move, coming from the inner need for moving, a characteristic of younger children was found to be lower. A significant decrease in the physical activity was observed for girls; among girls aged 11 years, every fifth girl declared satisfactory or high everyday-physical activity, while among those aged 15 years, only every tenth did so (26, 27).

Physical education classes are important in helping youth attain sufficient levels of physical activity. Bergier et al. assessed the physical activity levels of adolescents aged 16–18 years. They reported that the participation of adolescents in physical exercise classes was a factor that conditioned the level of total physical activity and that dismissing adolescents from physical exercise classes, especially adolescent girls, was common (28). In this study, boys were generally more physically active than girls. Overall, girls in other surveys were less physically active than boys, and more specifically they participated more in individual sports but less in team sports (29–32). In the study of obese teenagers, it was indicated that 29.1% girls and 21.1% boys did not regularly take part in physical education classes or did not participate at all. When compared to students with a proper body mass index, obese girls and boys more rarely exercised regularly during classes of physical education, which is consistent with previous research (33). The percentage of obese girls dismissed permanently from classes was twofold higher than girls with a proper body mass index (33).

Our study confirms the fact that obese students (42.3%) less willingly undertake any physical activity after school in comparison with students with proper BMI. Eutrophic students (44.6% girls and 69.7% boys) exercise more frequently and systematically than students with overweight and obesity (35.9% girls and 57.7% boys). According to the Australian study, physical activity after school was associated with lower than elevated BMI for boys and girls (34).

Some studies showed reductions in physical activity in adolescents (35) and the inverse association between total physical activity and overweight (36). According to some US studies, the reasons for low physical activity among young people included lack of time, usually due to doing homework, lack of motivation to move, lack of well-equipped sports facilities in the neighborhood, not safe enough neighborhood, bad weather, lack of appropriate equipment, and no company to do exercises together (37). HBSC study suggests that most adolescents spend two or more hours daily on computer games and two or more hours hanging on the internet (38). According to the survey of lifestyle risk factors Factores de Riesgo en ESColares (FRESC) among secondary school students (13–19 years old) (39), about half students watch television for ≥2 hours daily during weekdays. Boys reported playing videogames for ≥2 h/weekday more often than girls did (14.6% and 1.5%, respectively). Near 68.2% of boys and 61.7% of girls reported using computer for ≥2 hours/weekday. In our study, most adolescents exceeded the recommendations of 2 hours/day of watching TV and all students preferred to use a computer for ≥2 hours/day than to watch TV for ≥2 hours/day. Several studies report a positive relationship between longer period of watching TV, decreased physical activity, and obesity and overweight in children (10, 40–42). Watching TV influences the type and amount of food eaten by adolescents. Increased consumption of fats, sweets, salty snacks, fast-food, and decreased amount of fruit and vegetables were reported in children and adolescents spending free time in front of the TV or computer screen (43–45).

## Conclusion

A specific pattern of nutritional habits, that is, decreased meal frequency and skipping meals in adolescents were associated with overweight and obesity. This study also showed a high prevalence of insufficient physical activity levels and exposure to sedentary behaviors among adolescents. Girls presented lower physical activity than boys. The problem of obesity during growth requires systematic monitoring and suggests the need for prevention and change of nutritional habits early in childhood. It is essential for public health initiatives to promote diet and physical activity guidelines in adolescence.

## Ethical considerations

Ethical issues (Including plagiarism, informed consent, misconduct, data fabrication and/or falsification, double publication and/or submission, redundancy, etc.) have been completely observed by the authors.
